# Subclinical effects of remote ischaemic conditioning in human kidney transplants revealed by quantitative proteomics

**DOI:** 10.1186/s12014-020-09301-x

**Published:** 2020-11-02

**Authors:** Adam M. Thorne, Honglei Huang, Darragh P. O‘Brien, Marco Eijken, Nicoline Valentina Krogstrup, Rikke Norregaard, Bjarne Møller, Rutger J. Ploeg, Bente Jespersen, Benedikt M. Kessler

**Affiliations:** 1 Nuffield Department of Surgical Sciences and Oxford Biomedical Research Centre, University of Oxford, Oxford, UK; 2 Target Discovery Institute, Nuffield Department of Medicine, University of Oxford, Oxford, UK; 3 Department of Renal Medicine, Aarhus University Hospital, Aarhus, Denmark; 4 Department of Clinical Medicine, Aarhus University, Aarhus, Denmark; 5 Department of Renal Medicine, Rigshospitalet, Copenhagen, Denmark; 6 Department of Clinical Immunology, Aarhus University Hospital, Aarhus, Denmark

**Keywords:** CONTEXT clinical trial, Remote ischaemic conditioning, Acute phase proteins, ELISA, Proteomics, Mass spectrometry, Kidney transplantation

## Abstract

**Background:**

Remote ischaemic conditioning (RIC) is currently being explored as a non-invasive method to attenuate ischaemia/reperfusion injuries in organs. A randomised clinical study (CONTEXT) evaluated the effects of RIC compared to non-RIC controls in human kidney transplants.

**Methods:**

RIC was induced prior to kidney reperfusion by episodes of obstruction to arterial flow in the leg opposite the transplant using a tourniquet (4 × 5 min). Although RIC did not lead to clinical improvement of transplant outcomes, we explored whether RIC induced molecular changes through precision analysis of CONTEXT recipient plasma and kidney tissue samples by high-resolution tandem mass spectrometry (MS/MS).

**Results:**

We observed an accumulation of muscle derived proteins and altered amino acid metabolism in kidney tissue proteomes, likely provoked by RIC, which was not reflected in plasma. In addition, MS/MS analysis demonstrated transient upregulation of several acute phase response proteins (SAA1, SAA2, CRP) in plasma, 1 and 5 days post-transplant in RIC and non-RIC conditions with a variable effect on the magnitude of acute inflammation.

**Conclusions:**

Together, our results indicate sub-clinical systemic and organ-localised effects of RIC.

## Introduction

Remote ischaemic conditioning (RIC) has been propagated as a therapeutic strategy to protect organs against ischaemia/reperfusion injury (IRI). Within the setting of kidney transplantation, organ ischaemia is inevitable, and the resultant IRI can initiate a multitude of changes in the recipient, ranging from depletion of oxygen and nutritional supplies to mechanical tissue disruption, oedema and infiltration of immune cells [1]. IRI-inflicted tissue damage is complex, involving a variety of mechanisms that are not yet fully understood. Key to this is the production of reactive oxygen species (ROS) by anaerobic metabolism in response to hypoxic stress, a drop in cellular pH, and a rapid depletion of ATP. This constitutes a highly damaging combination that results in varying levels of cellular injury and ultimately leading to organ dysfunction. Upon reperfusion, immune cells accumulate at the site of injury releasing a myriad of pro-inflammatory molecules, propagating cellular injury and subsequently promoting apoptotic pathways [2, 3]. The damaging effects of IRI are especially apparent in kidney transplantation, triggering a range of pathophysiological cascades that can result in increased incidence of primary non-function (PNF) or delayed graft function (DGF) and episodes of acute rejection and graft fibrosis may follow, which all affect graft survival.

To combat the detrimental effects of transplant IRI, an intervention of remote ischaemic pre- or post-conditioning accompanying the expected period of ischaemia and reperfusion has been suggested to attenuate the magnitude of tissue damage [4, 5] in animal experiments, demonstrating beneficial effects and a reduction of IRI [6–10]. Also, proteomic analysis of plasma in RIC models found measureable changes of the acute inflammatory response [11, 12]. At the same time, mixed results have been observed, revealing no difference in levels of tubular damage caused by IRI after combined or separate pre- and post-conditioning [6, 13, 14]. More recently, the results of a randomised clinical trial in kidney transplantation (CONTEXT) were reported, applying RIC (preconditioning) to recipients of deceased donor kidneys. This multicentre trial was sufficiently powered, however, was not able to detect any improvement in its primary end point and secondary clinical transplant outcomes [15, 16]. As transplanted patients have various comorbidities and clinical parameters often lack granularity without being sensitive enough to identify more subtle molecular mechanisms possibly involved in potential beneficial effects of RIC, we decided to search for the presence of subclinical changes that could contribute to a systemic response resulting from the RIC regime administered. By analysing a cohort of sequentially obtained samples collected during the CONTEXT trial, we sought to determine whether RIC induced changes in recipient plasma and kidney tissue at the molecular level, using mass spectrometry based proteomics and enzyme linked immunosorbent assay (ELISA).

## Materials and methods

### CONTEXT patient cohort and RIC protocol

**Fig. 1:**
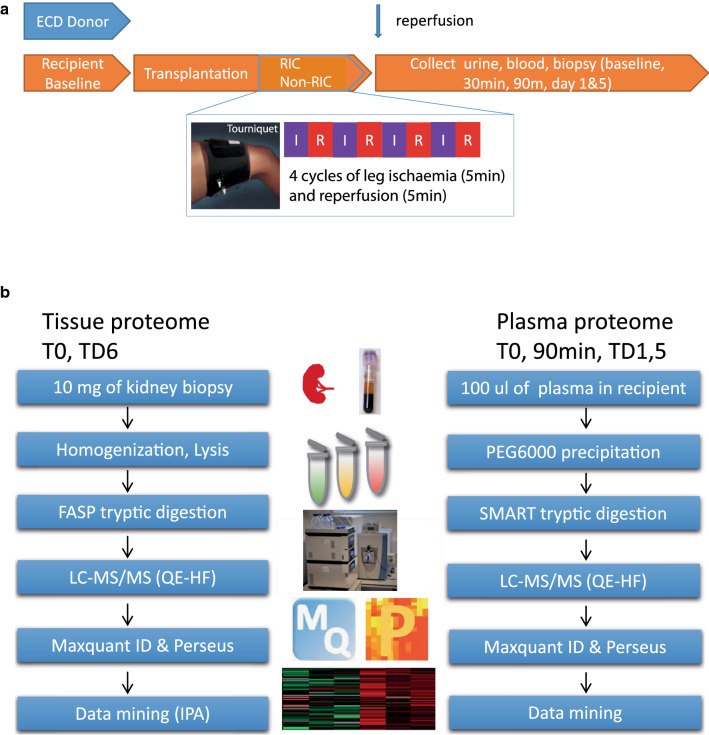
CONTEXT clinical trial sample collection and proteomics analysis workflows. Sample collection timeline of urine, blood and kidney tissue biopsy material (**a**). Proteomic workflows for kidney tissue (left panel) and plasma (right panel) (**b**)

The CONTEXT trial concerned 225 included transplant recipients and evaluated effects of RIC, induced by episodes of obstruction to arterial flow in the leg on the opposite side of where the transplant was implanted using a tourniquet (4 × 5 min) on the thigh prior to kidney reperfusion [15]. Trial registration: NCT01395719. Registered 14 July 2011, https://clinicaltrials.gov/ct2/keydates/NCT01395719. Measurements were compared to the non-RIC in a control group of recipients. Kidneys from the same donor were randomised to either RIC or non-RIC in a paired design. Recipients of deceased donor kidneys enrolled in CONTEXT were randomised to receive either treatment, or a sham procedure blinded to the physicians and surgeons involved. The RIC protocol consisted of four cycles of leg ischaemia (5 min) and reperfusion (5 min) at the thigh on the side opposing that used for transplantation. Blood samples were collected at baseline (T0), 30 min, 90 min, day 1 and day 5, respectively (Fig. 1a). Biopsies were taken 30 min after graft reperfusion (0-biopsy) and at day 6 [15]. The patient demographics ensured equal clinical characteristics between both arms, including a subgroup of 6 kidney pairs for LC–MS/MS analysis, and a cohort of 72 RIC/74 non-RIC donor recipient pairs containing samples at all measured time points for validation (Table 1) [17].

**Table 1:** CONTEXT patient cohorts for the MS discovery and validation studies

Patient cohort for the MS discovery study^a^			Patient cohort for validation study		
Recipient	RIC (n = 6)	Non-RIC (n = 6)	Recipient	RIC, n = 72	Non-RIC, n = 74
Gender			Gender		
Male	5	5	Male	46 (64%)	44 (59%)
Female	1	1	Female	26 (36%)	30 (51%)
Age, median (range)	52.2 (43.6–60.5)	48.0 (30.1–70.3)	Age, median (range)	57.3 (49.7–64.8)	60.8 (49.4–65.9)
Dialysis mode			Dialysis mode		
Pre-emptive	3	4	Preemptive	13 (18%)	15 (20%)
HD	2	1	HD	40 (56%)	42 (57%)
PD	1	1	PD	18 (25%)	16 (22%)
			HD + PD	1 (1%)	1 (1%)
Number of transplant			Number of transplant		
First	6	6	First	63 (88%)	65 (88%)
Retransplant	0	0	Retransplant	9 (13%)	9 (12%)
Original renal disease			Original renal disease		
Glomerulopathy	2	1	Glomerulopathy	20 (28%)	15 (20%)
ADPKD	2	2	ADPKD	15 (21%)	15 (20%)
Diabetes mellitus	1	1	Diabetes mellitus	8 (11%)	8 (11%)
Vascular/hypertension	0	1	Vascular/hypertension	7 (10%)	9 (12%)
Reflux/obstructive	0	0	Reflux/obstructive	3 (4%)	2 (3%)
Other	0	1	Other	6 (8%)	7 (9%)
Unknown	1	0	Unknown	13 (18%)	18 (24%)
Comorbidity			Comorbidity		
Diabetes mellitus	1	1	Diabetes mellitus	12 (17%)	14 (19%)
Hypertension	5	5	Hypertension	62 (86%)	69 (93%)
Total HLA-A, D, DR mismatches			Total HLA-A, B, DR mismatches		
0	0	0	0	3 (4%)	6 (8%)
1–2	0	0	1–2	15 (21%)	18 (24%)
3–4	5	6	3–4	47 (65%)	35 (47%)
5–6	1	0	5–6	7 (10%)	15 (20%)
Immunosuppression at discharge			Immunosuppression at discharge		
Tacrolimus	6	6	Tacrolimus	63 (88%)	72 (97%)
Mycophenolate mofetil	6	6	Mycophenolate mofetil	70 (97%)	74 (100%)
Corticosteroids	6	6	Corticosteroids	66 (92%)	72 (97%)
			Cyclosporine	7 (10%)	2 (3%)
			None (graft loss)	2	

Patient cohort for the MS discovery study^a^			Patient cohort for validation study		
Donor	RIC (n = 6)	Non-RIC (n = 6)	Donor	RIC, n = 72	Non-RIC, n = 74
Gender			Gender		
Male	4	4	Male n (%)	39 (54%)	41 (55%)
Female	2	2	Female n (%)	33 (46%)	33 (45%)
Age, median (range)	61.5 (55–72)	61.5 (55–72)	Age, median (range)	58.5 (50.5–65.5)	56.5 (49–63)
Paired kidneys	6	6	Paired kidneys	28 (39%)	28 (38%)
			Single kidneys	44 (61%)	46 (62%)
DBD	6	6	DBD	67 (93%)	69 (93%)
			DCD	5 (7%)	5 (7%)
Cause of death			Cause of death, DBD (n = 67 and 69)		
Cerebrovascular insult	5	5	Cerebrovascular insult	42 (63%)	43 (62%)
			Cerebral anoxia	15 (22%)	17 (25%)
Trauma	1	1	Trauma	9 (13%)	9 (13%)
			Benign cerebral neoplasm	1 (1%)	0
			Cause of death, DCD (n = 5 and 5)		
			Cerebrovascular insult	2	2
			Cardiac disease	1	2
			Trauma	0	1
			Other	2	0
Preservation solution			Preservation solutions		
Custodiol	5	5	Custodiol	56 (78%)	52 (70%)
UW	1	1	UW	15 /21%)	20 (27%)
			Other	1 (1%)	2 (3%)
			Warm ischemia time DCD, min	20 (15–25)	18 (14–25)
Cold ischemia time (h) Median (range)	12.3 (5.5–26.2)	13.5 (8.4–21.0)	Cold ischemia time (h) DBD + DCD		
			n > 24 h	13.4 (3.8)	13.9 (4.9)
			Missing data	1	4
				1	1
Total surgery time (h) Median (range)	2.1 (1.7–2.3)	2.3 (1.3–2.6)	Total surgery time (h) Median (range)	2.4 (2.0–2.8)	2.5 (2.0–3.0)
			Missing data	1	0
Remuzzi score, median (range)			Remuzzi score, median (range)		
All biopsies	2.5 (1–7) n = 6	3.5 (2–6) n = 6	All biopsies (n = 61 and 63)	2 (1–3)	2 (1–4)
6 glom	2.5 (1–7) n = 6	3.5 (2–6) n = 6	6 glom (n = 56 and 55)^b^	2 (1–3)	2 (1–4)
10 glom	3 (2–4) n = 3	3.5 (2–4) n = 4	10 glom (n = 41 and 42)^b^	2 (1–3)	2 (1–4)
			Missing data^c^	11 (15%)	11 (15%)

Data are mean (standard deviation), n (%), or median (interquartile range). Remuzzi scores on biopsies taken 30 min after reperfusion of the graft (baseline-biopsy)

Patient demographics and intraoperative data in RIC and non-RIC groups: left side two columns are the exploratory groups and right side two columns are the validation groups. Data are mean (standard deviation), n (%), or median (interquartile range). Remuzzi scores on biopsies taken 30 min after reperfusion of the graft (baseline-biopsy)

*HD* haemodialysis;* PD* peritoneal dialysis,* ADPKD* autosomal dominant polycystic kidney disease,* DBD* donation after brain death,* DCD* donation after circulatory death,* glom* glomeruli,* RIC* remote ischaemic conditioning,* UW* University of Wisconsin solution

^a^Samples used for the discovery cohort are included in the validation cohort

^b^Score if including only biopsies with minimum 6 or 10 glomeruli in the analysis

^c^Biopsies were either not performed or insufficient

### Sample preparation and analysis by mass spectrometry

For this in-depth study to detect any underlying molecular changes due to RIC our identifier cohorts consisted of six pairs of recipients (Table 1). We have analysed tissue and plasma samples from these six kidney pairs (6 RIC and 6 control), at 2 and 4 time points, respectively. Pairs of kidneys from the same donor where the recipients were as similar as possible, both had all samples taken including the day 6 biopsy and had no fevers, rejections or serious events within the first two weeks after transplantation. These samples were representative of the CONTEXT patient cohort. Detailed information can be found in Table 1 and [15].

### Tissue proteomics

Approximately 10 mg of kidney tissue (n = 6 per group, Table 1) was lysed in RIPA buffer (Pierce) using a bead beater homogenizer (Precellys). A BCA assay was performed and 50 µg of total protein from each sample was taken for Filter Aided Sample Preparation (FASP) digestion. In short, 30 kDa filters (Millipore) were equilibrated with 8 M urea buffer and spun through. For protein reduction, 50 µg of tissue lysate was added to 100 µL of 20 mM dithiothreitol (DTT) and incubated for 30 min at room temperature (RT). Samples were alkylated in 100 µL of 100 mM iodoacetamide (IAA) and incubated for 30 min at RT and in the dark. The samples were centrifuged and buffer exchanged with 8 M urea twice, and then with 50 mM ammonium bicarbonate three times. Trypsin was added at an enzyme:protein ratio of 1:30 (50 µL of 0.3 µg trypsin), and incubated overnight at 37 °C. The following day, the filters were inverted, centrifuged, and the digested peptides collected with washing steps of 0.5 M NaCl, ensuring maximum yield. The digested peptides were desalted using SOLAµ™ cartridges, as per the manufacturer’s instructions. Eluted samples were dried using a vacuum concentrator (Speedvac, Eppendorf) and resuspended in buffer A, consisting of 98% MilliQ-H_2_O, 2% acetonitrile (ACN) and 0.1% formic acid (FA). Samples were stored at − 20 °C until analysis by mass spectrometry (MS).

### Plasma proteomics

One hundred microliter of plasma were precipitated by adding PEG 6000 to final concentration of 12% in order to deplete abundant plasma proteins. Depleted plasma protein concentrations (n = 6 per group, Table 1) were determined using a BCA assay (Pierce) and digested using a Thermo Scientific™ SMART Digest™ Kit. Briefly, 50 µg of protein was added to 150 µL of the proprietary SMART digestion buffer and loaded into a SMART digestion tube containing immobilized trypsin beads. Samples were incubated at 70 °C and 1400 rpm for 2 h on an Eppendorf Thermomixer® C. Supernatants were collected by centrifugation at 2500×*g* for 5 min. The samples were desalted using SOLAµ™ solid phase extraction plates (Thermo Scientific, UK). Following washes of 0.1% trifluoroacetic acid (TFA) and elution in 65% ACN, samples were dried and resuspended for MS analysis as described above.

### Analysis by mass spectrometry

Liquid chromatography tandem mass spectrometry (LC–MS/MS) analysis was performed using a Dionex Ultimate 3000 nano-ultra high pressure reversed-phase chromatography system coupled on-line to a Q Exactive High Field (HF) mass spectrometer (Thermo Scientific) as described previously [18]. In brief, samples were separated on an EASY-Spray PepMap RSLC C18 column (500 mm × 75 µm, 2 µm particle size; Thermo Fisher Scientific) over a 60 min gradient of 2–35% ACN in 5% dimethyl sulfoxide (DMSO), 0.1% FA, and the flow rate was ~ 250 nL/min. The mass spectrometer was operated in data-dependent analysis (DDA) mode for automated switching between MS (MS1) and MS/MS (MS2) acquisition. Full MS survey scans were acquired from 400–2000 m*/z* at a resolution of 60,000 at 200 m*/z* and the top 12 most abundant precursor ions were selected for high collision energy dissociation (HCD) fragmentation. The resolution of MS2 fragment ion detection was also set to 15,000.

### ELISA assays for SAA1 and CRP

To validate proteomic findings, ELISA was used to detect and quantify SAA1 (R&D Systems, SAA1 DuoSet) levels in plasma of patients from the exploratory as well as the validation cohort where samples from all time points were available (n = 143, Table 1). Plates were coated overnight with capture antibody at RT and blocked with Reagent Diluent for 1 h at RT. Diluted plasma samples (100 µL) were incubated with the capture antibody for 2 h, followed by the detection antibody for 2 h, and finally, the HRP and substrate solution for 20 min, all at RT. Stop solution was added and the plate analysed using a TECAN plate reader at a wavelength of 540 nm, and with a second reading at 450 nm to account for plate aberration. In addition, we quantified CRP protein levels using an automated Simple Plex ELISA (Ella, Protein Simple) according to the manufacturer’s instructions.

### Measurements of S-troponin and S-myoglobin

In plasma from the whole CONTEXT cohort [15], ELISA assays were used to quantify levels of S-troponin T (Roche, Troponin T-high sensitive) and S-myoglobin (ADVIA Centaur XPT Immunoassay System) in plasma at baseline (TD0), 90 min and post-operative day 1 (TD1) according to the manufacturer’s instructions.

As data are mixed with some paired data (when kidney pairs were included) the following statistical method has been used: A repeated measurement analysis of variance was applied for the analysing S-troponin T, S-myoglobin [15]. A linear mixed regression effects model was used to compare log transformed outcomes between the intervention groups, with intervention and centre as fixed effects, and donor as random effect (Additional file 1: Table S1).

### Proteomics data analysis

**Fig. 2:**
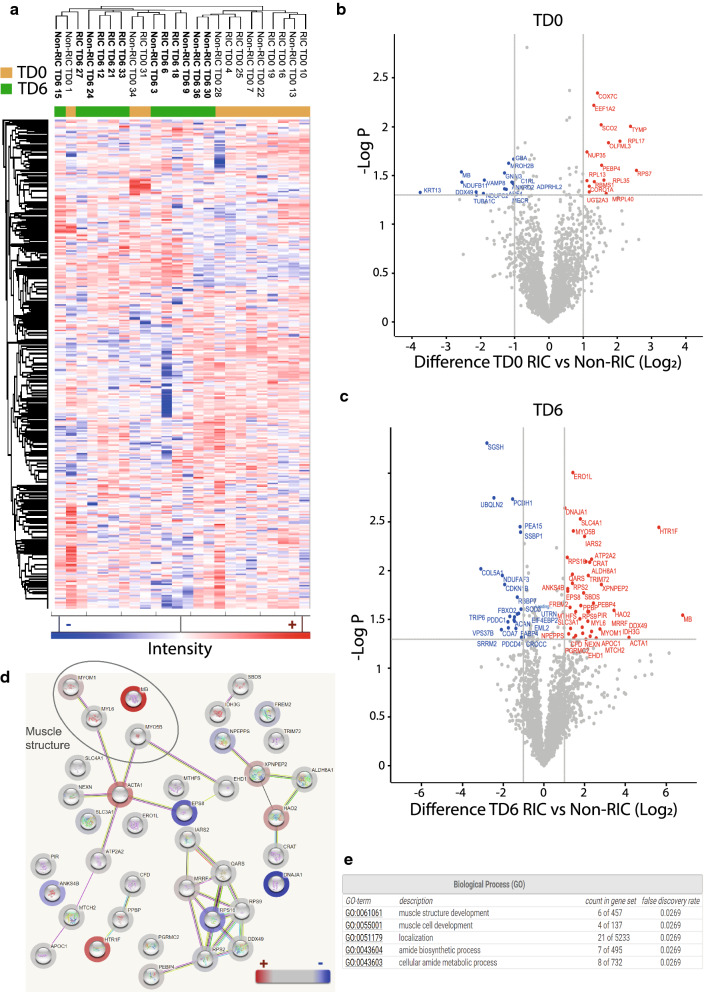
RIC versus non-RIC kidney tissue proteome profiles. Hierarchical clustering of kidney tissue proteome profiles using Euclidian distance (**a**). Volcano plot showing differential protein expression between RIC and non-RIC at baseline (TD0) (**b**) and between RIC and non-RIC 6 days post-transplant (TD6) (**c**). X-axis: protein level difference indicated by log2 fold change, Y-axis: statistical significance indicated by -log10 (p-value). Overall, 30 and 62 proteins, for A and B respectively, have a fold change greater than 2 (at a *p*-value < 0.05). Up-regulated proteins are highlighted in red, while their down-regulated counterparts are coloured blue. **d** Biological pathways reflected by elevated proteins in kidney tissues at day 6 (TD6), RIC versus non-RIC (Table S2). Halos in red / blue indicate the abundance levels of proteins used in this analysis. **e** Enriched biological process (GO-terms) from proteins shown in **d**

**Fig. 3:**
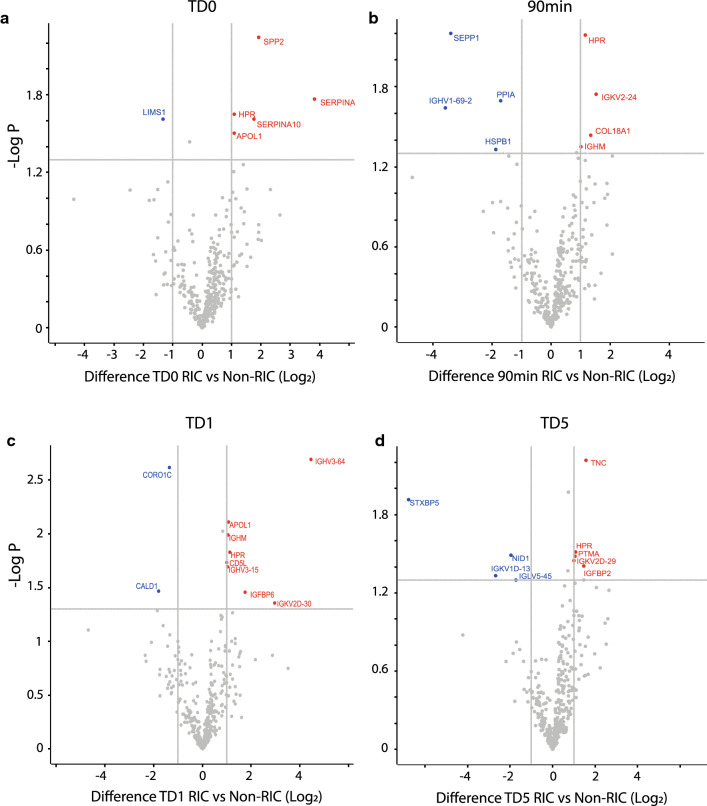
Plasma proteomes affected by RIC as compared to non-RIC. Volcano plot showing plasma protein level changes between: RIC and non-RIC at baseline (0hrs) (**a**), RIC and non-RIC at 90 min (90 min) (**b**), RIC and non-RIC at 1 day post-transplant (TD1) (**c**), RIC and non-RIC 5 days post-transplant (TD5) (**d**). X-axis: protein level difference indicated by log2 fold change, Y-axis: statistical significance indicated by – log10 (*p*-value). In total, 6, 8, 10, and 9 proteins, have a fold change greater than 2 (at a *p*-value < 0.05) for **a**, **b**,** c **and** d** respectively. Up-regulated proteins are highlighted in red and down regulated proteins are coloured blue

MS raw data was searched using MaxQuant software (v1.3.1). The search parameters used were as follows: trypsin with two missed cleavages allowed, oxidation (M) and deamidation (N, Q) were set as variable modifications and carbamidomethylation (C) as a fixed modification. The data was searched against human protein sequences using the UPR_homoSapiens_20141015 fasta file along with the corresponding decoy revert database. Only unique and razor peptides were used for quantitation. Identified peptides were processed using Perseus software (v1.5.4.1), which filtered out contaminant and false positive identifications (decoys). Label-free quantitation (LFQ) values are extracted from MS spectra in MaxQuant using the MaxLFQ algorithm [19]. Accurate protein abundance was calculated from the sum of all peptide intensities (the maximum detector peak intensity over the peptide elution profile). MaxLFQ normalised the data to account for the variability of quantifiable peptides across all samples. The data were log_2_ transformed, and replicates were grouped into RIC and controls. The missing values were replaced using a strategy that considers normal distribution of imputed values. Heat maps were created in Perseus using the Pearson correlation algorithm applied after converting values to Z scores. Hierarchical cluster analysis was performed on all the proteins identified by LC–MS/MS and visualised as heat maps. For the proteomics data analysis (Additional file 2: Tables S2, Additional file 3: S3) and the generation of the volcano plots (Figs. 2,3, Additional file 4: Fig. S1), we applied a parametric RIC Student t-test (assuming a normal distribution of the quantitative mass spectrometry data) to compare between RIC and control samples as well as between the different time points as indicated in the individual figure legends (log2 of the difference in total intensities per protein) and using a Permutation FDR based correction for multiple testing [20]. The mass spectrometry proteomics data have been deposited to the ProteomeXchange Consortium via the PRIDE [21] partner repository with the dataset identifier PXD019284. Biological pathway enrichment analysis was performed using STRING Protein–protein interaction networks & functional enrichment analysis (https://string-db.org/). Enriched kidney tissue protein subsets in D5 RIC versus non-RIC (Additional file 2: Table S2) were used to create graphs and pathways shown in Fig. 2d, e. Enriched plasma protein subsets in D6 RIC and non-RIC (Additional file 3: Table S3) were used to create graphs and pathways shown in Additional file 6: Figure S3A and B.

## Results

To explore whether RIC causes any changes at the organ tissue level, we performed an initial discovery analysis, in which a subset of kidney pairs (n = 6 subjects per arm) were selected to represent the entire cohort (Table 1). Kidney tissue biopsy and recipient plasma samples were subjected to protein extraction, digestion and analysis by nano-liquid chromatography tandem mass spectrometry (LC–MS/MS) analysis as described in Materials & Methods (Fig. 1b). LC–MS/MS identified 2731 and 378 protein groups for kidney tissue and plasma, respectively.

**Fig. 4:**
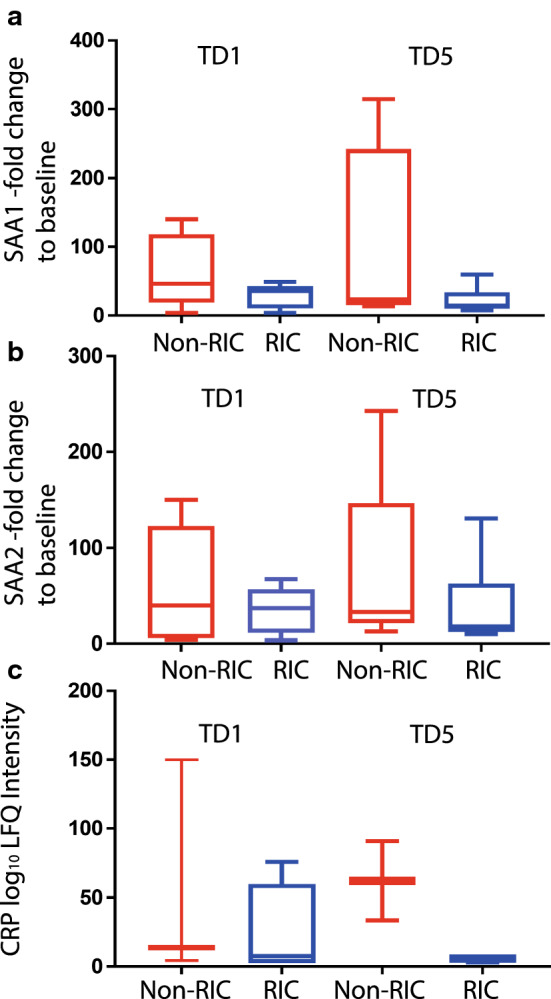
Reduced levels of acute phase proteins SAA1/2 and CRP after RIC. Box-plot representation of peptide precursor mass ions assigned to SAA1 (**a**), SAA2 (**b**) and C-reactive protein (CRP) as measured by LC–MS/MS (**c**). LFQ intensities detected in RIC vs non-RIC at 1 and 5 days post-transplant were compared and normalised to baseline (TD0) levels (**a**,** b**) or displayed as Log_10_ values (**c**)

**Fig. 5:**
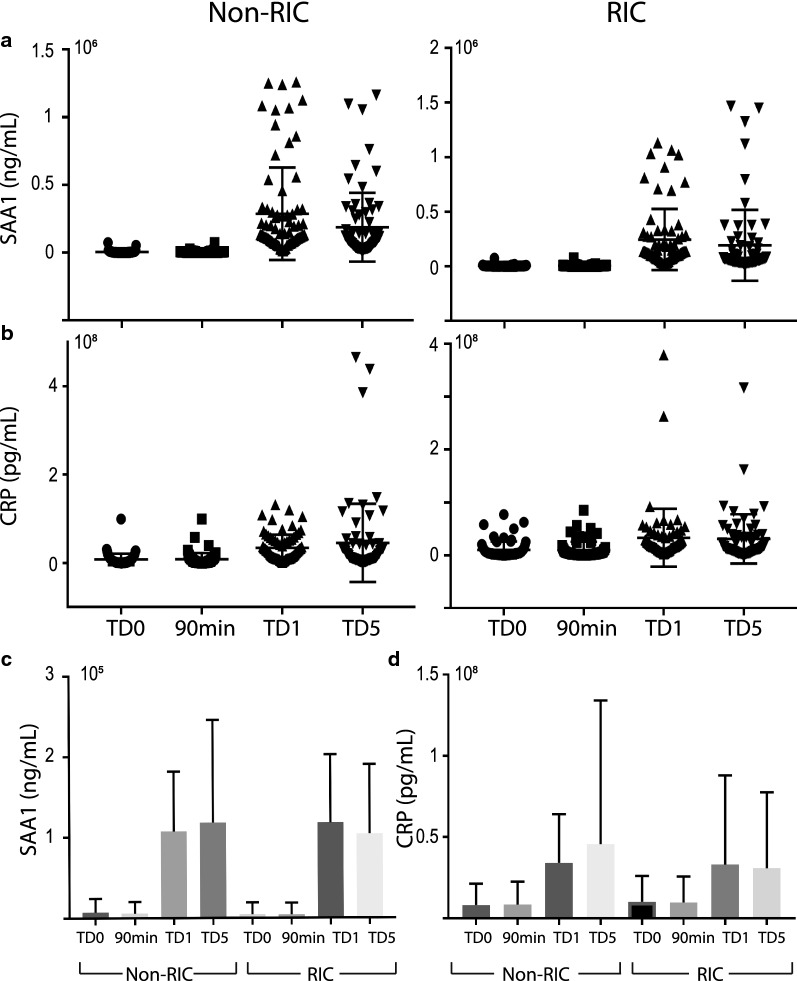
Effect of RIC on SAA1 and CRP levels in the blood of CONTEXT patients. ELISA validation of SAA1 (**a** dot plot**, c** bar graph) and CRP **(b** dot plot**, d** bar graph) at baseline (TD0), 90 min, 1 (TD1) and 5 days (TD5) post-transplant in both RIC and non-RIC. The y-axis represents quantified levels of target in ng/mL for **a**, **c**, and pg/mL for **b**, **d**

Analysis of individual proteins in the kidney tissue proteomes revealed 44 proteins that were differentially expressed with at least a twofold change in abundance between RIC and non-RIC groups (Additional file 2: Table S2). Following analysis of the biopsies of the donor kidney pairs, from which one organ was implanted in a RIC and the other one in a non-RIC recipient, overall kidney tissue proteome profiling analysed by clustering analysis distinguishes between sample specimens taken at baseline (TD0) versus day 6 (TD6) (Fig. 2a). However, the analysis does not discriminate between RIC and non-RIC (Fig. 2a). At the same time, it was found that when comparing RIC and non-RIC at 6 days post-transplant, kidney proteomes from RIC patients had an accumulation of muscle proteins or proteolytic fragments thereof, such as myosin heavy chain 2 (MYH2), myosin light chain 3 (MYL3), myoglobin (MB) and synemin (SYNM) (Fig. 2b, c), also reflected by biological pathway analysis (Fig. 2d, e). This finding was not substantiated by the detection of elevated P-myoglobin and troponin in RIC group derived patient plasma at TD0 (baseline), 90 min and TD1 (day 1) by ELISA when compared to non-RIC patients, using all patients of the CONTEXT trial (Additional file 1: Table S1). This suggests intact kidney filtering function of muscle degradation products resulting from RIC. In addition, we also detected up-regulated proteins in RIC as compared to non-RIC after 6 days (TD6), such as 5-hydroxy-tryptamine receptor 1F (HTR1F), hydroxyacid oxidase 2 (HAO2), Xaa-pro-aminopeptidase (XPNPEP2), that can be assigned to altered amino acid metabolism (Fig. 2c–e, Additional file 2: Table S2). The plasma proteome was also profiled by tandem mass spectrometry, showing that antioxidant proteins including PRDX2 and HPR were upregulated transiently at 90 min and 1 day after transplantation in RIC. In addition, acute phase response proteins were found to be up-regulated in RIC, but to a lesser extent as compared to non-RIC groups after 1 day and 5 days, in particular the acute phase components Apo1, SAA1, SAA2 and CRP (Fig. 3, Additional file 4: Fig. S1, Additional file 5: Fig. S2, Additional file 6: Fig. S3A, B and Additional file 3: Table S3). The number of differentially expressed proteins and magnitude of increase, in particular for SAA1, SAA2 and CRP, was higher in non-RIC versus RIC (Fig. 4 and Additional file 4: Fig. S1). For the purpose of validation, the full patient cohort, where samples were available at all time points, was tested using ELISA assays against SAA1, SAA2 and CRP. Analysis confirmed an increase in these acute phase components over time, but the initially observed difference between the RIC and non-RIC groups was not confirmed within the larger patient cohort (Fig. 5 and Additional file 5: Fig. S2).

## Discussion

In the past decades, claims have been made that Remote Ischaemic Conditioning (RIC) will reduce IRI and enhance the viability and function of kidneys after transplantation [4, 5]. To substantiate evidence in a clinical context a well-designed and sufficiently powered clinical multicentre trial in kidney transplantation was carried out. Despite using a bespoke and previously tested RIC regimen, this trial in kidney transplantation did not demonstrate an effect on clinical outcomes after transplantation of deceased donor kidneys when recipients had been exposed to the RIC [15, 16]. Following this negative study, the more principle question arose as to whether the chosen RIC regimen had actually triggered any subtle subclinical response at all, especially in the presence of an obvious heterogeneity with diverse and multimorbid uraemic patients. To detect any systemic and/or end organ changes related to the RIC administered, we have analysed plasma and kidney tissue collected from representative transplant recipients in both arms of the trial. First, our proteomics study reveals that RIC provoked several effects at the molecular level. First, we observed that kidney tissue contains muscle proteins after RIC. Second, enzymes involved in amino acid metabolism were increased in RIC kidney tissue, suggesting metabolic alteration in accordance with previous studies in human and animal models [1–3]. Third, in our exploratory group of patients, plasma from RIC patients demonstrated a lesser increase in levels of transient acute phase response proteins when compared with non-RIC. All these effects, however, appear to be temporary and limited. They were not confirmed in our validation samples and no correlation was found with any changes in clinical outcomes in the CONTEXT trial [15, 16].

In general, RIC induced effects in vivo have variable outcomes. Another RIC study conducted in a pig kidney transplant model did result in slightly improved short-term GFR and renal plasma perfusion [6] without effecting immunological sentinel cells, such as dendritic cells, in the blood [22] or general inflammation [23]. It is likely that in a clinical study when RIC is applied in humans, subclinical changes maybe very subtle and challenging to detect. Mass spectrometry based analysis of kidney biopsies as described here revealed several noticeable features at a molecular level that could potentially be exploited to further optimise the RIC regimen, although recent studies have not pointed to positive clinical effects of RIC [15, 24]. We observed accumulated muscle proteins in kidney tissue from RIC patients, most likely derived from muscle injury inflicted by a repeated mechanical obstruction to the arterial flow in the leg opposite the transplant using a tourniquet or to the pressure of the tourniquet on the muscles [25]. Higher levels or myoglobin and troponin in the RIC group in kidney tissue may be attributed to the response of muscle to the induction of hypoxia. In particular, upregulation of myoglobin, via calcineurin and NFAT mediated transcription, has been widely associated with muscle contraction in combination with response to hypoxia as well as being an important indicator of muscle damage [26, 27]. However, it is not clear if the change in these proteins could contribute to protective mechanisms of RIC in the recipient, or whether it is purely a result of muscle damage induced by applying the tourniquet. Myoglobin is excreted primarily by the kidney, and carries a risk of acute kidney injury which was however not seen in the CONTEXT study [15]. Although a rise of myoglobin was not demonstrated in the plasma of RIC patients the accumulation in the kidneys with their glomerular filtration of plasma could be potentially harmful.

Proteomics analysis also suggests that RIC leads to altered energy metabolism in kidney tissue 6 days after transplantation. This is consistent with other reports in experimental models that suggested reduced IRI in kidney after RIC, although it was mainly attributed to reduced oxidative stress [1, 28, 29], while re-oxygenation leads to similar benefits [30, 31].

Plasma proteomics in this small discovery cohort (n = 6 per group) revealed a transient effect of RIC on protein expression, particularly in relation to the attenuation of acute phase inflammatory response proteins 1 and 5 days after transplantation, predominantly reflected by SAA1, SAA2 and CRP. Interestingly, complement factor D (CFD), detected in plasma and kidney tissue, was reduced in non-RIC as compared to RIC in tissue (Additional file 2: Table S2, Additional file 3: Table S3). However, we were unable to validate the RIC mediated effect on the magnitude of the acute phase response in all samples available within the CONTEXT cohort using bespoken ELISAs. The apparent discrepancy between the quantitative mass spectrometry results and the ELISA assays for SAA1, SAA2 and CRP tested are most likely due to the different cohort size for discovery (MS; n = 6/6, rlC vs CTRL) versus validation (ELISA; n = 72/74, rlC vs CTRL), but possibly also due to the fact that mass spectrometry and proteomics measure proteolytic (tryptic) peptide fragments, whereas ELISA uses antibody-based recognition of epitopes within intact proteins. It is therefore feasible that multiple protein isoforms may not all be recognized equally well by antibody-based ELISA, but are quantified differentially at the peptide level. As an example, SAA1 is originally secreted from the liver as an ~ 11 kDa protein into plasma, but then becomes rapidly processed proteolytically to ~ 8.5 kDa [32, 33], which could alter quantitation by different methods.

Generally, an explanation for the challenges found in translating RIC from an animal model to human setting may be due to the method of RIC induction itself. In this CONTEXT study, RIC was achieved using a tourniquet around the thigh, whereas in pigs [15], a clamp was placed on the distal aorta; below the renal arteries, but before the aortic bifurcation. Although ischaemic cycles remained the same in both regimens, the pig model presents a more invasive and aggressive strategy, possibly resulting in a more amplified, and therefore physiologically noticeable, response. Another explanation may be that pigs simply respond to such injury in a different way than humans, which may not be comparable. Furthermore, the human patients were much older with comorbidity and various medical treatments than the infant pigs that were studied [15].

## Conclusions

Taken together, this study focused on the detection of subclinical molecular effects as a result of the targeted intervention against IRI using a bespoke RIC regimen in kidney transplantation. While the randomised clinical trial had not found any difference in transplant kidney function and clinical outcomes between treated and control groups, we wondered whether the RIC had triggered a response at all on the level of the proteome in samples from both groups. We appreciate that previous studies in controlled large animal models have demonstrated beneficial effects of targeted RIC strategies. However, our results from the in-depth analysis of samples from the clinical CONTEXT trial including proteomics data suggest that the applied regimen of RIC is not appropriate for mounting an effective molecular response. This finding may implicate that RIC will not exert a positive effect on pathways involved in injury/repair mechanisms in the human kidney when compared to results in other organs such as the heart, where a clinically beneficial response to RIC has been reported, but not confirmed in a recently published larger study [24]. In combination with the negative results from the CONTEXT clinical trial, it must be concluded that the RIC strategy applied will provoke some subclinical molecular changes of transient nature, but is not effective and does not elicit a significant and detectable response, either systemically, or in the target organ itself.

## Supplementary information

**Additional file 1: Table S1.** S-myoglobin and S-troponin ELISA levels in Plasma. Values represent levels of S-troponin and S-myoglobin in patient plasma samples at baseline (TD0), 90 mins and 1 day post-transplant (TD1) and day 5 (TD5) as measured by ELISA. Estimated median s-troponin t ng/L and estimated median s-myoglobin μg/L, at baseline, 90 min and post-operative day one. No significant differences were found between the two groups.

**Additional file 2: Table S2.** Altered kidney proteome tissue profiles in RlC vs non-RlC. Table showing kidney tissue protein and gene names that demonstrate an >2-fold change (Log2) and *p*-value <0.05 (Log) as measured by LC-MS/MS. RIC vs non-RIC baseline (TD0) and day 5 (TD5) are displayed. Highest positive to highest negative fold change are indicated from red to green, respectively.

**Additional file 3: Table S3.** Altered plasma proteome profiles in RlC vs non-RlC. Table showing plasma protein gene names that demonstrate an > 2-fold change (Log2) and *p*-value <0.05 (Log) as measured by LC-MS/MS. Baseline (TD0) vs 90min, day 1 (TD1) and day 5 (TD5) in RIC and Non-RIC conditions are displayed. Highest positive to highest negative fold change are indicated from red to green, respectively.

**Additional file 4: Figure S1.** Plasma proteomes affected by RIC as compared to non-RIC. Volcano plots showing plasma proteome derived protein level changes at baseline (TD0) vs 90 min (A), 1 day (TD1) and 5 day (TD5) in both RIC and non-RIC groups. X-axis: protein level difference indicated by log_2_ fold change, Y-axis: statistical significance indicated by –log_10_ (*p*-value). Up-regulated proteins (>2-fold change, p-value <0.05) are highlighted in red and down regulated proteins are coloured blue.

**Additional file 5: Figure S2.** RIC affects SAA1 and CRP levels in the blood of CONTEXT patients. ELISA validation of SAA1 and CRP at baseline (TD0), 90 min, 1 (TD1) and 5 (TD5) days post-transplant in both RIC and non-RIC. The y-axis represents quantified levels of target in ng/ml for SAA1 and pg/ml CRP.

**Additional file 6: Figure S3.** Elevated acute phase proteins in plasma of RIC and non-RIC patients. Biological pathways reflected by elevated proteins in patient plasma in RIC (**A**) and non-RIC (**B**) conditions analysed using STRING (see ”Materials and methods” section).

## Data Availability

Data available on request to the CONTEXT Data Access Committee, Bente Jespersen, Aarhus University Hospital, (bentjesp@rm.dk). It is not possible to anonymise data sufficient for public access according to Danish Data Protection Regulations. Experimental data presented in this manuscript, in particular ELISA, are available upon request by the authors (contact email: benedikt.kessler@ndm.ox.ac.uk). The mass spectrometry proteomics data is publicly available via the ProteomeXchange Consortium PRIDE with the dataset identifier PXD019284.
