# Supplementary material for: Subclinical effects of remote ischaemic conditioning in human kidney transplants revealed by quantitative proteomics
Source: Clin Proteomics. 2020 Nov 2;17:39. doi: 10.1186/s12014-020-09301-x (PMC7607690; doi:10.1186/s12014-020-09301-x)
Supplement: Supplementary file 1 — Additional file 1: Table S1. S-myoglobin and S-troponin ELISA levels in Plasma. Values represent levels of S-troponin and S-myoglobin in patient plasma samples at baseline (TD0), 90 mins and 1 day post-transplant (TD1) and day 5 (TD5) as measured by ELISA. Estimated median s-troponin t ng/L and estimated median s-myoglobin μg/L, at baseline, 90 min and post-operative day one. No significant differences were found between the two groups. [file 12014_2020_9301_MOESM1_ESM.pdf]

Table S1: Troponin T and myoglobin levels in plasma

	RIC	Sham RIC	p
Troponin T			
Baseline, N=217	37 (95% CI 33-42)	38 (95% CI 33-43)	0.84
90 min, N=212	38 (95% CI 32-42)	33 (95% CI 29-38)	0.26
Day 1, N=214	33 (95% CI 28-39)	34 (95% CI 29-40)	0.71
Myoglobin			
Baseline, N=218	197 (95% CI 179-218)	202 (95% CI 183-222)	0.75
90 min, N=214	268 (95% CI 241-297)	258 (95% CI 233-286)	0.57
Day 1, N=214	346 (95% CI 297-402)	389 (95% CI 336-451)	0.25

Estimated median s-troponin t ng/L and estimated median s-myoglobin µg/L, at baseline, 90 min and post-operative day one. No significant differences were found between the two groups.
