# Supplementary material for: Subclinical effects of remote ischaemic conditioning in human kidney transplants revealed by quantitative proteomics
Source: Clin Proteomics. 2020 Nov 2;17:39. doi: 10.1186/s12014-020-09301-x (PMC7607690; doi:10.1186/s12014-020-09301-x)
Supplement: Supplementary file 2 — Additional file 2: Table S2. Altered kidney proteome tissue profiles in RlC vs non-RlC. Table showing kidney tissue protein and gene names that demonstrate an >2-fold change (Log2) and p-value <0.05 (Log) as measured by LC-MS/MS. RIC vs non-RIC baseline (TD0) and day 5 (TD5) are displayed. Highest positive to highest negative fold change are indicated from red to green, respectively. [file 12014_2020_9301_MOESM2_ESM.pdf]

Table S2: Altered kidney tissue proteome profiles in RIC vs non-RIC

Gene Names	Protein Names	Baseline (RIC vs non-RIC)		Day 6 (RIC vs non-RIC)	
		LOG (P-value)	Log2 (fold change)	LOG (P-value)	Log2 (fold change)
MB	Myoglobin			1.54	6.82
HTR1F	5-hydroxytryptamine receptor 1F			2.45	5.63
ACTA1	Actin, alpha skeletal muscle			1.32	4.18
HAO2	Hydroxyacid oxidase 2			1.59	3.44
XPNPEP2	Xaa-Pro aminopeptidase 2			1.86	2.83
MYOM1	Myomesin-1			1.40	2.76
MRRF	Ribosome-recycling factor, mitochondrial			1.31	2.57
PEBP4	Phosphatidylethanolamine-binding protein 4			1.67	2.42
ATP2A2	Sarcoplasmic/endoplasmic reticulum calcium ATPase 2			2.12	2.35
MTCH2	Mitochondrial carrier homolog 2			1.38	2.34
APOC1	Apolipoprotein C-I			1.33	2.27
ALDH8A1	Aldehyde dehydrogenase family 8 member A1			2.09	2.24
MTHFS	5-formyltetrahydrofolate cyclo-ligase			1.58	2.19
TRIM72	Tripartite motif-containing protein 72			1.95	2.18
PIR	Pirin			1.58	2.15
DDX49	Probable ATP-dependent RNA helicase DDX49			1.48	2.15
NEXN	Nexilin			1.30	2.07
CRAT	Carnitine O-acetyltransferase			2.09	2.07
IARS2	Isoleucine--tRNA ligase, mitochondrial			2.35	2.00
SBDS	Ribosome maturation protein SBDS			1.77	1.95
CFD	Complement factor D			1.36	1.90
IDH3G	Isocitrate dehydrogenase [NAD] subunit			1.42	1.88
PPBP	Platelet basic protein			1.65	1.81
SLC4A1	Band 3 anion transport protein			2.53	1.79
MYL6	Myosin light polypeptide 6			1.51	1.77
RPS9	40S ribosomal protein S9			1.58	1.57
EHD1	EH domain-containing protein 1			1.33	1.56
PGRMC2	Membrane-associated progesterone receptor component 2			1.31	1.47
MYO5B	Unconventional myosin-Vb			2.41	1.45
ERO1L	ERO1-like protein alpha			3.01	1.42
QARS	Glutamine--tRNA ligase			1.96	1.40
RPS2	40S ribosomal protein S2			1.87	1.39
SLC3A1	Neutral and basic amino acid transport protein rBAT			1.41	1.31
FREM2	FRAS1-related extracellular matrix protein 2			1.62	1.29
NPEPPS	Puromycin-sensitive aminopeptidase			1.35	1.22
ANKS4B	Ankyrin repeat and SAM domain-containing protein 4B			1.81	1.17
EPS8	Epidermal growth factor receptor kinase substrate 8			1.79	1.16
RPS16	40S ribosomal protein S16			2.14	1.16
DNAJA1	DnaJ homolog subfamily A member 1			2.64	1.02
VPS37B	Vacuolar protein sorting-associated protein 37B			1.32	-1.01
CROCC	Rootletin			1.30	-1.05
PDCD4	Programmed cell death protein 4			1.32	-1.11
SOD3	Extracellular superoxide dismutase [Cu-Zn]			1.61	-1.12
SSBP1	Single-stranded DNA-binding protein, mitochondrial			2.39	-1.15
PEA15	Astrocytic phosphoprotein PEA-15			2.45	-1.18
FBXO2	F-box only protein 2			1.56	-1.25
RBBP7	Histone-binding protein RBBP7			1.73	-1.31
EIF4EBP2	Eukaryotic translation initiation factor 4E-binding protein 2			1.56	-1.31
FABP4	Fatty acid-binding protein, adipocyte			1.41	-1.38
PDDC1	Parkinson disease 7 domain-containing protein 1			1.53	-1.44
EML2	Echinoderm microtubule-associated protein-like 2			1.49	-1.45
ACAN	Aggrecan core protein;Aggrecan core protein 2			1.50	-1.46
PCDH1	Protocadherin-1			2.73	-1.53
TRIP6	Thyroid receptor-interacting protein 6			1.53	-1.68
SRRM2	Serine/arginine repetitive matrix protein 2			1.41	-1.74
UTRN	Utrophin			1.48	-1.77
CDKN1B	Cyclin-dependent kinase inhibitor 1B			1.86	-1.92
NDUFAF3	NADH dehydrogenase [ubiquinone] 1 alpha subcomplex assembly factor 3			1.96	-2.03
COA7	Cytochrome c oxidase assembly factor 7			1.39	-2.08
UBQLN2	Ubiquilin-2			2.75	-2.44
SGSH	N-sulphoglucosamine sulphohydrolase			3.31	-2.80
COL5A1	Collagen alpha-1(V) chain			2.02	-3.10
RPS7	40S ribosomal protein S7	1.55	2.56		
TYMP	Thymidine phosphorylase	2.00	2.39		
RPL17	60S ribosomal protein L17	1.85	2.08		
OLFML3	Olfactomedin-like protein 3	1.84	1.75		
MRPL40	39S ribosomal protein L40, mitochondrial	1.32	1.67		
RPL35	60S ribosomal protein L35	1.45	1.61		
PEBP4	Phosphatidylethanolamine-binding protein 4	1.61	1.54		
SCO2	Protein SCO2 homolog, mitochondrial	2.02	1.52		
COX7C	Cytochrome c oxidase subunit 7C, mitochondrial	2.34	1.42		
RBMS1;RBMS3	RNA-binding motif, single-stranded-interacting protein 1	1.44	1.33		
EEF1A2	Elongation factor 1-alpha 2	2.22	1.31		
UGT2A3	UDP-glucuronosyltransferase 2A3	1.33	1.18		
CORO1A	Coronin-1A;Coronin	1.39	1.18		
NUP35	Nucleoporin NUP53	1.74	1.12		
RPL13	60S ribosomal protein L13	1.45	1.11		
ADPRHL2	Poly(ADP-ribose) glycohydrolase ARH3	1.43	-1.02		
GBA	Glucosylceramidase	1.67	-1.03		
C1RL	Complement C1r subcomponent-like protein	1.41	-1.04		
ANKRD2	Ankyrin repeat domain-containing protein 2	1.43	-1.08		
MROH2B	Maestro heat-like repeat-containing protein family member 2B	1.63	-1.18		
MECR	Trans-2-enoyl-CoA reductase, mitochondrial	1.36	-1.25		
ARF4	ADP-ribosylation factor 4	1.37	-1.30		
GNAI3	Guanine nucleotide-binding protein G(k) subunit alpha	1.53	-1.30		
VAMP8	Vesicle-associated membrane protein 8	1.45	-1.88		
NDUFC2	NADH dehydrogenase [ubiquinone] 1 subunit C2	1.32	-1.92		
TUBA1C	Tubulin alpha-1C chain	1.30	-2.12		
DDX49	Probable ATP-dependent RNA helicase DDX49	1.33	-2.13		
NDUFB11	NADH dehydrogenase [ubiquinone] 1 beta subcomplex subunit 11, mitochondrial	1.43	-2.55		
MB	Myoglobin	1.54	-2.55		
KRT13		1.32	-3.77		

Relative protein abundance: ++ + - - -
