# Supplementary material for: Subclinical effects of remote ischaemic conditioning in human kidney transplants revealed by quantitative proteomics
Source: Clin Proteomics. 2020 Nov 2;17:39. doi: 10.1186/s12014-020-09301-x (PMC7607690; doi:10.1186/s12014-020-09301-x)
Supplement: Supplementary file 3 — Additional file 3: Table S3. Altered plasma proteome profiles in RlC vs non-RlC. Table showing plasma protein gene names that demonstrate an > 2-fold change (Log2) and p-value <0.05 (Log) as measured by LC-MS/MS. Baseline (TD0) vs 90min, day 1 (TD1) and day 5 (TD5) in RIC and Non-RIC conditions are displayed. Highest positive to highest negative fold change are indicated from red to green, respectively. [file 12014_2020_9301_MOESM3_ESM.pdf]

Table S3: Altered plasma proteome profiles in RIC vs non-RIC

Gene Name	Non-RIC 90min vs TD0		Non-RIC TD1 vs TD0		Non-RIC TD5 vs TD0		RIC 90min v T0		RIC TD1vs TD0		RIC TD5 vs TD0	
	-LOG (P value)	Log2 (fold change)	-LOG (P value)	Log2 (fold change)	-LOG (P value)	Log2 (fold change)	-LOG (P value)	Log2 (fold change)	-LOG (P value)	Log2 (fold change)	-LOG (P value)	Log2 (fold change)
SAA1			2.51	7.22	2.91	8.10			4.02	4.83	3.91	4.45
SAA2			2.97	7.02	3.48	7.02			3.64	4.78	3.24	4.62
CRP			4.19	5.88	3.65	5.12			2.21	4.47	1.51	4.24
SERPINA7					1.42	2.77						
F5			1.36	1.87	1.87	2.69						
YWHAZ					1.94	2.43					1.76	2.50
PLEK	1.62	1.40	1.96	2.20	2.03	2.39						
IGLC7					1.34	2.33	1.92	1.09				
TLN1					1.54	2.11						
RAP1B;RAP1A	2.34	1.69	2.10	1.73	2.90	2.06						
PPIA					1.62	2.03					1.84	1.90
FCGBP					2.58	1.91						
FLNA					1.36	1.77						
VCL					1.87	1.76					1.67	2.18
PFN1			1.46	1.71	2.21	1.65						
SERPINA3			2.68	1.20	4.54	1.64					3.78	1.42
ACTA1;ACTC1;ACTG2;ACTA2					1.56	1.45						
SAA2-SAA4;SAA4			1.44	1.17	1.84	1.35						
ACTG1;ACTB					1.85	1.22					1.71	1.12
ITIH3					1.93	1.17						
HP					2.96	1.04						
LRG1			3.67	1.03	4.00	1.03						
C9					3.10	1.02						
SIGLEC16					1.85	-1.02						
TNC					1.35	-1.14						
CFD			1.38	-1.32	1.90	-1.89						
MED1					1.57	-2.09						
FGA					1.42	-2.34						
PTGDS					1.54	-2.66						
CAP1			1.63	2.36							2.16	1.88
CD14			1.93	1.15								
GPX3			1.38	1.56							1.38	2.42
HBA1;HBA2			1.86	-1.48					2.08	-1.20		
HBB			2.91	-1.68					1.61	-1.14		
HBD			1.39	-1.22								
IGFBP6			2.38	-1.65								
ILK			1.43	1.98								
LBP			1.89	2.37								
NID1			3.10	1.84								
PLA2G7	2.20	2.75	1.54	2.07								
PLTP			1.73	1.40								
PRSS2			1.69	1.52								
TAGLN2			1.60	3.03								
TUBB1			1.49	1.47								
BTD	1.64	1.38										
REG3A							1.87	-2.06				
SEPP1							1.37	-2.33				
RNH1									1.47	1.80		
SOD1									1.52	1.52		
VWF									2.01	2.35	2.61	3.51
ACTN1;ACTN4											1.47	1.75
CEP290											1.66	-2.67
ENO1											1.46	1.39
FERMT3											2.01	1.91
FGL1											1.49	1.20
MYL9											1.56	2.47
PF4;PF4V1											1.76	1.43
ZYX											2.20	1.85

Relative protein abundance: ++ | + | - | - -
