# Supplementary figures and images for: Subclinical effects of remote ischaemic conditioning in human kidney transplants revealed by quantitative proteomics
Source: Clin Proteomics. 2020 Nov 2;17:39. doi: 10.1186/s12014-020-09301-x (PMC7607690; doi:10.1186/s12014-020-09301-x)

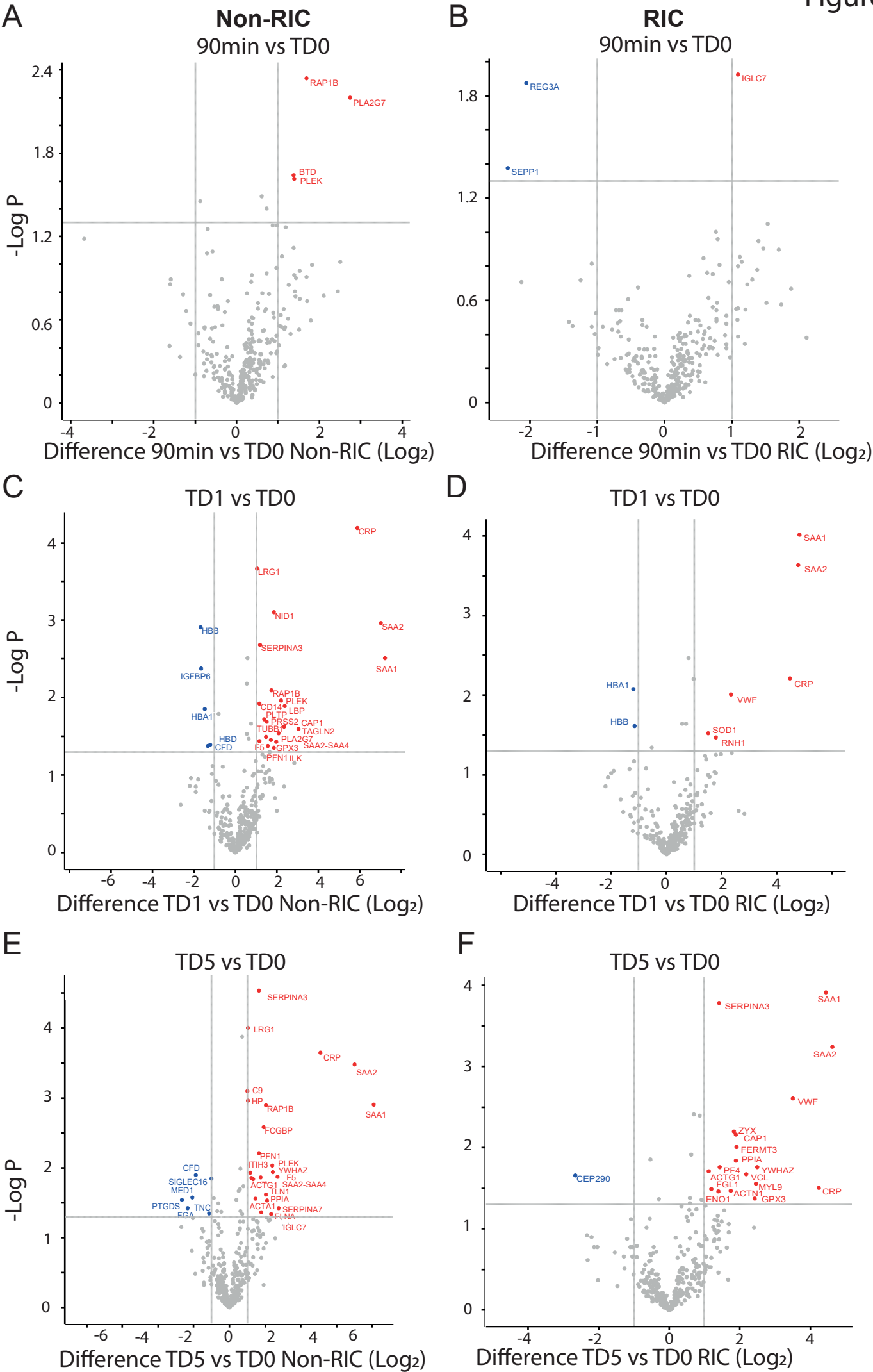

Supplement: Supplementary file 4 — Additional file 4: Figure S1. Plasma proteomes affected by RIC as compared to non-RIC. Volcano plots showing plasma proteome derived protein level changes at baseline (TD0) vs 90 min (A), 1 day (TD1) and 5 day (TD5) in both RIC and non-RIC groups. X-axis: protein level difference indicated by log2 fold change, Y-axis: statistical significance indicated by –log10 (p-value). Up-regulated proteins (>2-fold change, p-value <0.05) are highlighted in red and down regulated proteins are coloured blue. [file 12014_2020_9301_MOESM4_ESM.pdf]

Figure S2

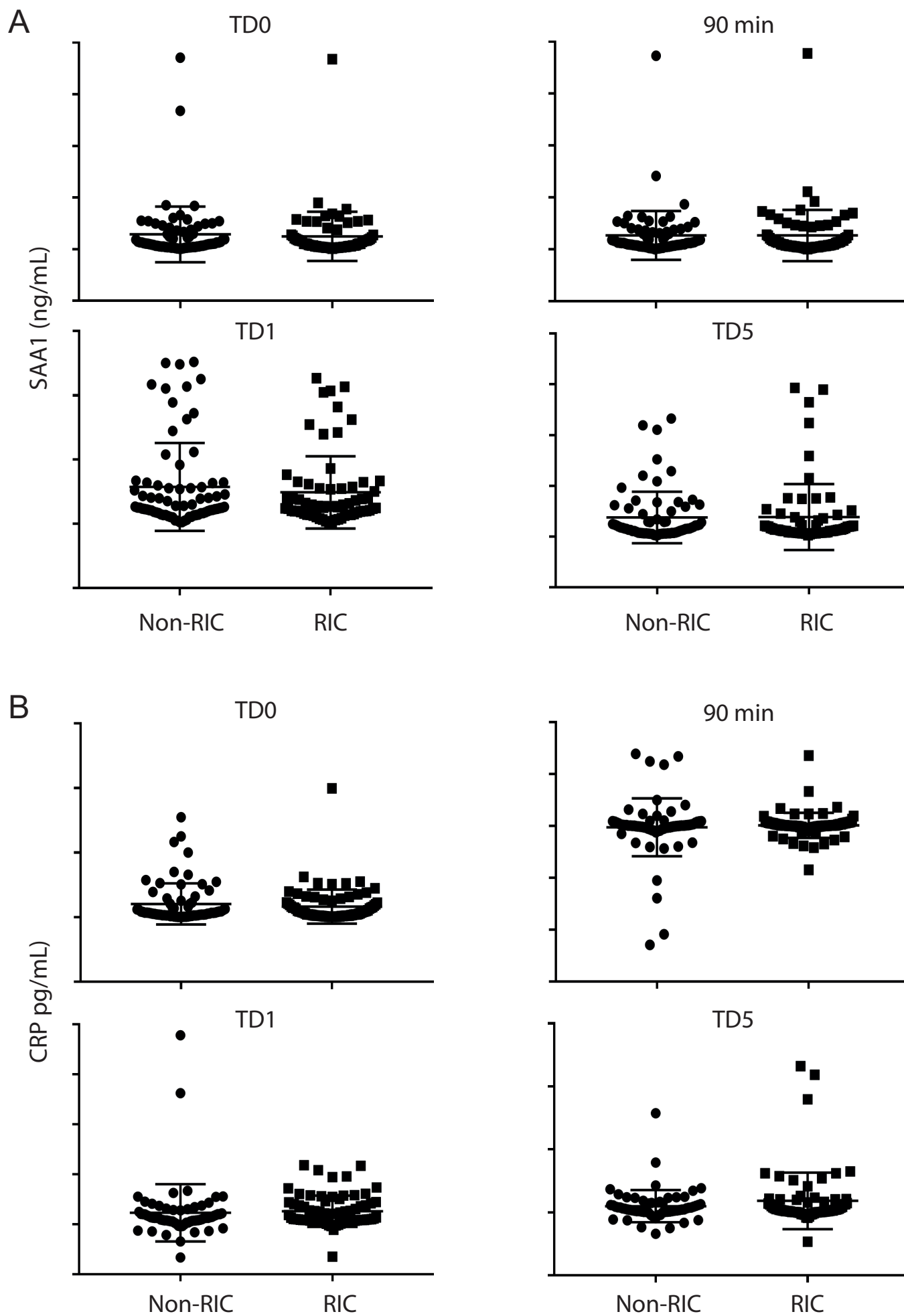

Supplement: Supplementary file 5 — Additional file 5: Figure S2. RIC affects SAA1 and CRP levels in the blood of CONTEXT patients. ELISA validation of SAA1 and CRP at baseline (TD0), 90 min, 1 (TD1) and 5 (TD5) days post-transplant in both RIC and non-RIC. The y-axis represents quantified levels of target in ng/ml for SAA1 and pg/ml CRP. [file 12014_2020_9301_MOESM5_ESM.pdf]
