# Supplementary material for: Subclinical effects of remote ischaemic conditioning in human kidney transplants revealed by quantitative proteomics
Source: Clin Proteomics. 2020 Nov 2;17:39. doi: 10.1186/s12014-020-09301-x (PMC7607690; doi:10.1186/s12014-020-09301-x)
Supplement: Supplementary file 6 — Additional file 6: Figure S3. Elevated acute phase proteins in plasma of RIC and non-RIC patients. Biological pathways reflected by elevated proteins in patient plasma in RIC (A) and non-RIC (B) conditions analysed using STRING (see ”Materials and methods” section). [file 12014_2020_9301_MOESM6_ESM.pdf]

Figure S3

A non-RIC

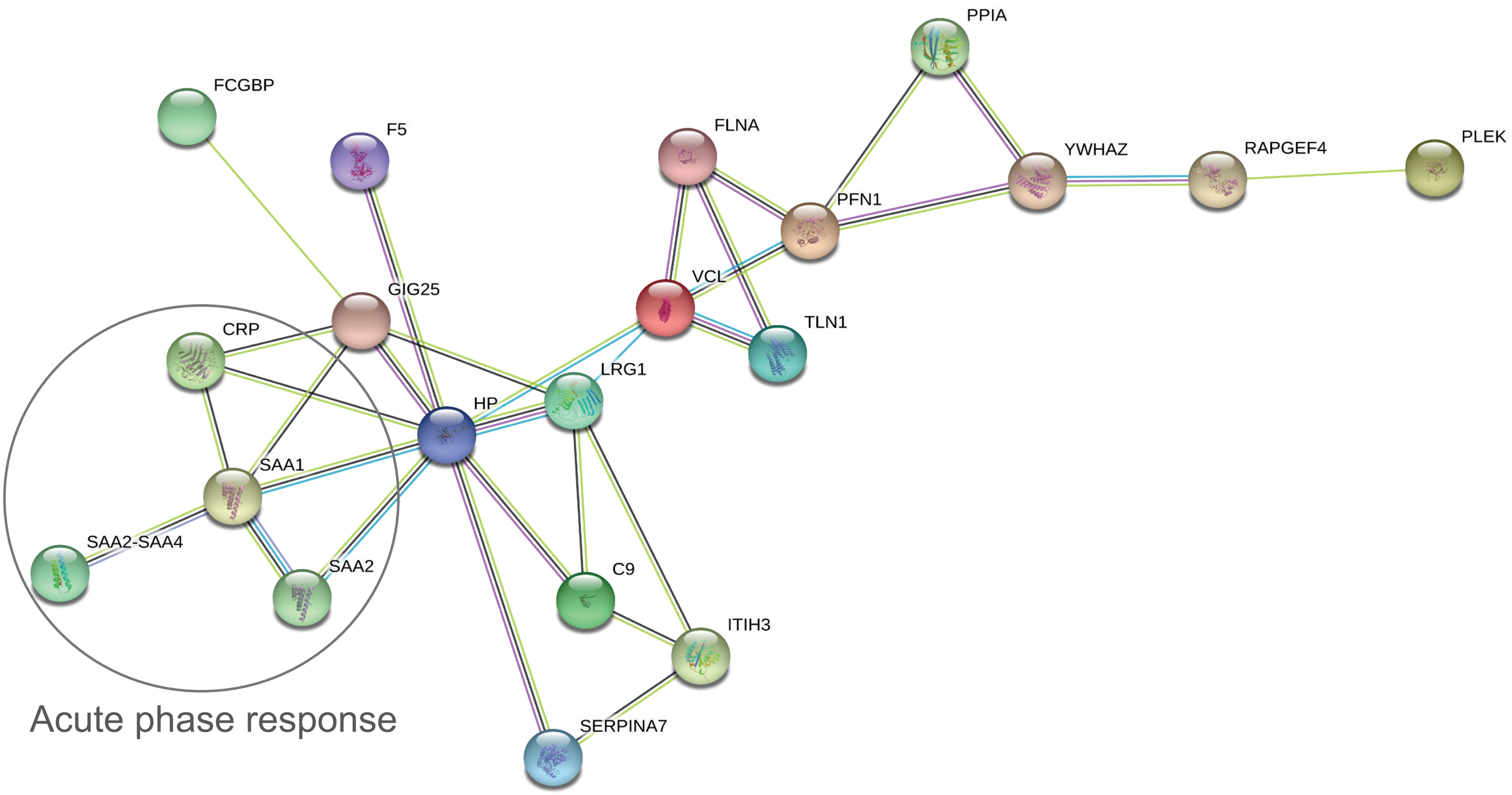

Biological Process (GO)			
GO-term	description	count in gene set	false discovery rate
GO:0045055	regulated exocytosis	11 of 691	1.33e-08
GO:0002576	platelet degranulation	7 of 129	2.19e-08
GO:0016192	vesicle-mediated transport	13 of 1699	1.31e-07
GO:0006953	acute-phase response	5 of 45	2.03e-07
GO:0006810	transport	15 of 4130	3.93e-05

B RIC

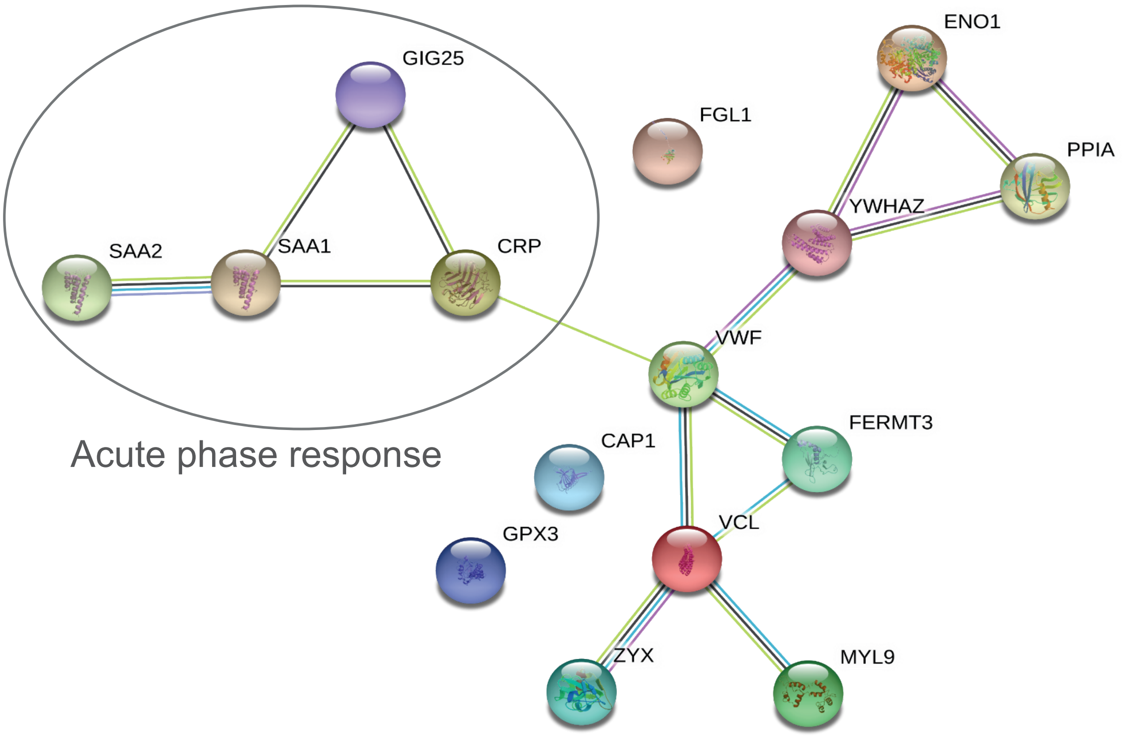

Biological Process (GO)			
GO-term	description	count in gene set	false discovery rate
GO:0006953	acute-phase response	4 of 45	3.27e-05
GO:0002576	platelet degranulation	4 of 129	0.00062
GO:0001775	cell activation	7 of 1024	0.00086
GO:0045055	regulated exocytosis	6 of 691	0.00089
GO:0031589	cell-substrate adhesion	4 of 162	0.00089
